# European Ash (*Fraxinus excelsior*) as a Functional Keystone Species Shaping Woodland Flora in the UK

**DOI:** 10.1002/ece3.73356

**Published:** 2026-04-02

**Authors:** Melanie Roach, Ben Raymond

**Affiliations:** ^1^ Department of Ecology and Conservation University of Exeter Penryn UK; ^2^ School of Geography and Environmental Science University of Southampton Southampton UK

**Keywords:** ash dieback, disease impacts, functional diversity, woodland conservation, woodland flora

## Abstract

Ash dieback disease, caused by the pathogenic fungus *Hymenoscyphus fraxineus*, is now widespread in the UK. The pathogen has caused substantial loss of European ash over recent decades, the long‐term consequence of which is complex to assess. While no higher plant species are exclusively associated with ash, widespread ash mortality is predicted to affect woodland floral biodiversity because of the functional traits uniquely associated with this species: namely the greater light penetration through the canopy, rapid foliar decomposition and nutrient cycling abilities. In woodlands where ash is frequent, vascular plant communities may undergo considerable compositional changes after loss of ash trees. At present little is known regarding how ash trees contribute to plant community and functional diversity at a fine scale across different woodland types. Using data from 1274 survey plots characterized in the Woodland Survey of Great Britain alongside plant functional trait data, we examined the local relationship between ash trees and plant community diversity and functional trait distribution. We show that ash trees are significantly associated with key plant functional traits and diversity indices and that this functional association is not typical of other dominant canopy species in the UK. Specific leaf area, nutrient and pH requirements and community diversity show significant correlations with ash basal area across various woodland types. Additionally, effects of ash appear to interact with soil pH resulting in a greater structural effect of ash upon plant community composition in lower pH soils. These findings support previous suggestions that ash functions as a keystone species with respect to nutrient cycling and plant community composition with potentially stronger influence on lower pH soils. Consideration should be given to these ecological roles when monitoring and addressing the impacts of widespread ash mortality from dieback disease across Europe.

## Graphical abstract

Ash trees have distinct ecological attributes—their canopy allows a high level of light to penetrate and their foliage decomposes rapidly. Here we used woodland survey data to explore if the prevalence of ash trees has an impact on associated plant communities at a fine spatial scale. Unusually among dominant tree species, ash affects plant community diversity and the functional traits shared by this community; surprisingly, these correlations are stronger on woodland soils with low pH.

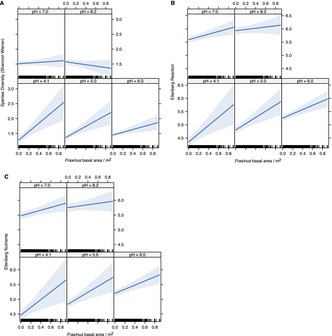

## 1 Introduction

Deciduous woodlands are the climax vegetation over much of the United Kingdom (UK) land area and can host a disproportionately rich amount of biodiversity (Peterken 1996; Rackham 2008). Woodlands are essential components of climate and environment‐regulating processes such as carbon sequestration, flood prevention, and nutrient cycling (Aerts and Honnay 2011; Mori et al. 2017) while providing economically important provisions in timber, wood products, and raw materials (Nesbitt et al. 2017; ONS 2025) alongside vital esthetic, health, and recreational benefits (O'Brien et al. 2014; Urquhart 2007). The UK's highly fragmented woodlands are vulnerable to a range of pressures including development, pollution, climate change, and increasingly, novel pests and pathogens (Carr 2021; Bebber et al. 2025). Introduced tree diseases and pests are a growing global problem in forests worldwide (Panzavolta et al. 2021; Stenlid et al. 2011; Liebhold et al. 1995), but the UK has seen a particularly steep rise of biotic threats in its woodlands and plantations (Stewart et al. 2026; Gilligan et al. 2013). In 1992, the UK had noted just two significant examples of novel tree pests and pathogens in the previous 30 years: Dutch elm disease and spruce bark beetle ( *Dendroctonus micans* ) (Kugelann 1794). The following three decades witnessed the arrival of no less than 19 notifiable or reportable pests or diseases of concern which are collectively capable of causing considerable damage to more than a dozen tree species (IPF 2012; Forest Research 2022).

Uniquely affecting select members of the *Fraxinus* genus, symptoms of the ash dieback pathogen were first reported in Poland in 1995 (Przybył 2002). The causal agent of the disease is an ascomycete fungus initially described in its anamorph (asexual) stage as *Chalara fraxinea* (Kowalski 2006) and later referred to as *Hymenoscyphus pseudoalbidus* (Queloz et al. 2011). It is now recognized as *Hymenoscyphus fraxineus* (Baral et al. 2014). It is not quite clear how *H. fraxineus* arrived in Europe, although the Manchurian Ash ( *Fraxinus mandshurica* Rupr.) has been suggested as a potential source (Zhao et al. 2012). *H. fraxineus* exists as a leaf litter decomposer on *F. mandshurica* without causing significant disease, likely due to co‐evolutionary host resistance (Nielsen et al. 2017).

*Hymenoscyphus fraxineus* spread extensively across mainland Europe from the 1990s onwards and crossed to the UK by 2012 (Pautasso et al. 2013a; Enderle et al. 2019) where it now affects a significant proportion of ash trees throughout the country. *H. fraxineus* infects European ash *Fraxinus excelsior* L. (hereafter referred to as ‘ash’) and other susceptible ash species with ascospores released in late spring to late summer from overwintered rachis on the forest floor (Gross et al. 2014). The primary infection route involves ascospores landing on leaves during summer, causing necrotic lesions which spread through the rachis and petiole into shoots and branches (Gross et al. 2014; Cleary et al. 2013). However, *H. fraxineus* may also bypass foliar infection by directly penetrating shoots via lenticels (Nemesio‐Gorriz et al. 2019), through the epidermis of young shoots (Mansfield et al. 2018), or by infecting roots from soil‐borne inoculum in waterlogged conditions (Fones et al. 2016; Baxter et al. 2023). The fungus colonizes woody tissue, extending through the stem to invade cambial and bark tissues (Schumacher 2011). While crown dieback is the most visible symptom, tree death commonly results from necrotic lesions at the stem and root collar, ultimately destabilizing the tree (Langer 2017; Chandelier et al. 2016; Baxter et al. 2023). Some mature trees can survive repeated crown infections for years, often producing epicormic shoots (McKinney et al. 2014) but disease progression is generally more rapid in saplings and younger trees (Nielsen et al. 2017).

Examination of mortality rates of ash trees on the European continent exposed to *H. fraxineus* for up to 20 years has led to predictions of 50%–75% mortality in established natural woodlands and up to 100% mortality in plantations. Across 36 surveyed patches, the highest maximum mortality found for trees was 72% and 82% for saplings (Coker et al. 2019). A recent analysis of long‐term mortality and crown condition monitoring data across 27 European countries found a mean survival probability of 0.51 after nearly 30 years of infection, which ranged from 0.2 to 0.86 across regional contexts (George et al. 2022). Genetic studies suggest some cautious optimism; between 1% and 5% of ash trees may be tolerant to *H. fraxineus* infection (Lobo et al. 2015) and experimental trials indicate that resistance to ash dieback has a heritable genetic component, with some families consistently producing more resilient offspring (Seidel et al. 2024). Furthermore, a comparison of the reproductive outcomes of healthy versus diseased ash trees in Denmark found the genes of healthy parent trees to be more abundant in sampled seeds and seedlings (Semizer‐Cuming et al. 2021), thus natural selection may ultimately favor resilient genotypes. Pollination studies further support this view showing that trees with lower disease severity contribute more pollen in ash growing in seed orchards and floodplains in Germany (Eisen et al. 2023). Breeding tolerance or resistance may be feasible (Clark and Webber 2017; Hultberg et al. 2020) but with breeding programmes still in early stages, the short to mid‐term expectation is that a considerable number of ash specimens will be lost. The progression and severity of infections across Europe suggest an epidemiological pattern where peak infection occurs in most areas at around 10–15 years after first observation (Vacek et al. 2017; Havrdova et al. 2017; Matisone et al. 2021).

Ash has some distinct functional characteristics which has led to it being proposed as a ‘keystone’ forest species (Agostinelli et al. 2021; Hultberg et al. 2020). Ash saplings in early growth stages are more shade‐tolerant than many other canopy species; regeneration is not significantly influenced by light availability and has been observed to be more successful under heterospecific canopies (Detsch et al. 2025) with denser, mixed stands favoring seedling emergence (Jochner‐Oette et al. 2021) although higher light conditions are required when saplings reach > 50 cm (Harmer et al. 2012). Ash may allow greater light penetration beneath the canopy due to specific leaf morphological traits and an open canopy structure (Thomas 2016). Within‐stand comparisons at the Hainich mixed forest (Hölscher 2004) found that ash consistently showed substantially lower leaf area indices (LAI) at lower canopy positions that were 24%–32% less than in sycamore, hornbeam and lime. Since LAI measures total leaf area per unit ground area, lower values directly imply less interception of incoming light. Ash also had the lowest Specific Leaf Area (SLA) of all species studied, likely reflecting its compound leaf architecture, in which significant biomass is invested in the rachis and petioles rather than foliage. Although young ash saplings are shade tolerant, adult trees show a distinct ontogenetic shift—become relatively light‐demanding and not developing a shade leaf layer seen in many other European canopy species. The most distinct functional trait of ash however is its rapid nutrient cycling ability (Wardle 1961; Thomas 2016). Ash leaf litter has a low C:N ratio and low lignin content and soils under ash have been found to have higher nitrogen and exchangeable Mg^2+^ and Ca^2+^ (Langenbruch et al. 2012) thus ash litter degrades swiftly, providing a rapid return of nitrates to forest soils. The rapid rate of decomposition of ash litter is not correlated with environmental factors such as soil biota and moisture (Jacob et al. 2009), which contrasts with other tree species. The relatively thin litter layer under ash is also associated with decreased acidifying ability (Ranger et al. 2002; Oostra et al. 2006). Ash can be associated with base rich clays and calcareous soils with neutral to alkaline conditions. However, it occurs over a wide range of soil types and soils with varying pH (Wardle 2013; Rodwell 1991; Hall et al. 2004). Together, these traits suggest ash creates a structurally and functionally distinct canopy; promoting nutrient cycling and transmitting more light to the woodland floor thus contributing to the diverse vernal ground flora characteristic of ash‐dominated NVC communities (Hall et al. 2004; Mitchell, Hewison, et al. 2016).

Ash dieback is predicted to affect woodland plant communities through the structural impact of losing a high volume of a major canopy species followed by loss of the enhanced light‐penetration qualities of ash. Ash‐dominant woodlands are predicted to undergo the most significant changes in the short to medium term; canopy gap creation following ash mortality will initially increase light levels, potentially assisting colonization by grasses and competitor species, with implications for forest regeneration (Mitchell, Hewison, et al. 2016; Erfmeier et al. 2019; Jochner‐Oette et al. 2021). Following canopy gap creation, the eventual replacement of ash by deeper shade‐casting species, such as sycamore, is likely (Needham et al. 2016; Detsch et al. 2025). Subsequent changes to litter composition and light regimes may impact nutrient cycling and significantly shift plant community assemblages (Mitchell, Bailey, et al. 2014; Mitchell, Beaton, et al. 2014; Mitchell, Hewison, et al. 2016). The colonizing species following loss of ash will partly determine later successional patterns and relate directly to the future biodiversity potential of affected sites (Needham et al. 2016). Additional biogeochemical impacts have been hypothesized which relate to the rapid nutrient cycling functions associated with ash trees (Mitchell, Bailey, et al. 2014; Mitchell, Beaton, et al. 2014; Mitchell, Pakeman, et al. 2016). The interrelationship of aboveground and belowground plant–soil interactions has been well described (Scheu 2005; Bardgett and Wardle 2010) and changes to leaf‐litter properties and soil nutrient regimes which may accompany loss of ash could also potentially affect vascular plant niches by altering soil properties. Disturbance and changes to the litter layer have been shown to induce longer term changes in plant, fungal and soil communities as well as nutrient inputs to forest streams (Sydes and Grime 1981; Sayer 2006; Smock and MacGregor 1988). Impacts of ash dieback are likely to be site‐specific due to differential severity of infection along climatic and soil gradients (Erfmeier et al. 2019; Turczański et al. 2020) and variability in host resistance (Prospero and Cleary 2017). The loss of principal tree species in forest ecosystems has been associated with large‐scale ecosystem changes in community composition and functioning elsewhere. In eastern North American forests, long‐term (> 50 years) structural change to stream channels occurred following large inputs of deadwood from American chestnuts affected by blight (Ellison et al. 2005), A 43% loss in eucalypts due to *Phytopthera cinnamoni* in Australia transformed the ground vegetation, previously dominated by sclerophyllous Xanthorrhoea species, to a sedge and rush‐dominated understorey (Weste 2003). (Mitchell, Hewison, et al. 2016; Needham et al. 2016; Erfmeier et al. 2019; Jochner‐Oette et al. 2021).

The extent to which ash trees influence soil and forest floor conditions and, by extension, plant community functional diversity, in both ash‐occasional and ash‐frequent woodlands is generally unknown (Pautasso et al. 2013b; Mitchell, Beaton, et al. 2014). Although very few species are uniquely associated with ash, we would expect ash to drive plant community changes by reason of its unique functional traits. The aim of this study is to examine how the functional diversity of plant communities is affected by ash composition in UK woodlands to aid assessment of the full range of consequences following widespread loss of ash in UK woodlands. Our approach was to use floristic data from the repeat Woodland Survey of Great Britain (Wood et al. 2015) undertaken in 2001–2003 to investigate potential relationships between tree species composition and plant community functional traits at a fine scale (plots within sites). Previous analyses of the 1971 Woodland Survey of Great Britain data assessed how floral composition is affected by regional and local factors and found that tree species composition had small effects relative to geo‐climactic factors (Corney et al. 2006). Nevertheless, that study was not designed to explore fine scale plot level variation where local conditions and plot characteristics may be more important. The woodland survey has recently been repeated and can provide insights into how ash dieback may have affected vegetation composition in its first 20‐year progression (Smart, Walker, et al. 2024). The 2001–2003 dataset however is more appropriate to address our specific research question concerning the long‐term functional influences of European ash on plant communities. The 2001–2003 survey preceded widespread ash dieback establishment in UK woodlands, providing a baseline relatively unaffected by this disease and thus enabling clearer inference of ash‐specific effects on community composition. Despite notable species losses between the 1971 and 2001 surveys, we considered the 2001 data more representative of long‐term conditions under ash; it has been observed that the 20th century had been particularly ‘kind to ash’ due to land management changes and gray squirrel introduction limiting competition from understorey species (Rackham 2014), potentially making ash less ubiquitous in earlier periods.

The hypothesis that ash influences plant community functional composition through its unique nutrient cycling attributes has not been tested. We hypothesize that the extent of ash in plots may correlate more prominently than other tree species with plant functional traits that are strongly coupled to light, nutrient and pH requirements. In addition, we hypothesize that ash, as a potential functional keystone, will correlate with increased species diversity. We would expect to see these relationships at the plot level across a range of woodland types including those where ash is not dominant or frequent. This study uses national woodland survey data and a multiple regression mixed modeling approach to explore how tree species composition affects plant community functional traits and community diversity at the plot scale, after accounting for site associated factors. To determine whether ash trees associate with distinctive plant functional trait values, our analysis includes the basal area of the six other most commonly occurring broadleaved species in the sample plots as a comparison.

## 2 Methods

### 2.1 Study Sites

**FIGURE 1:**
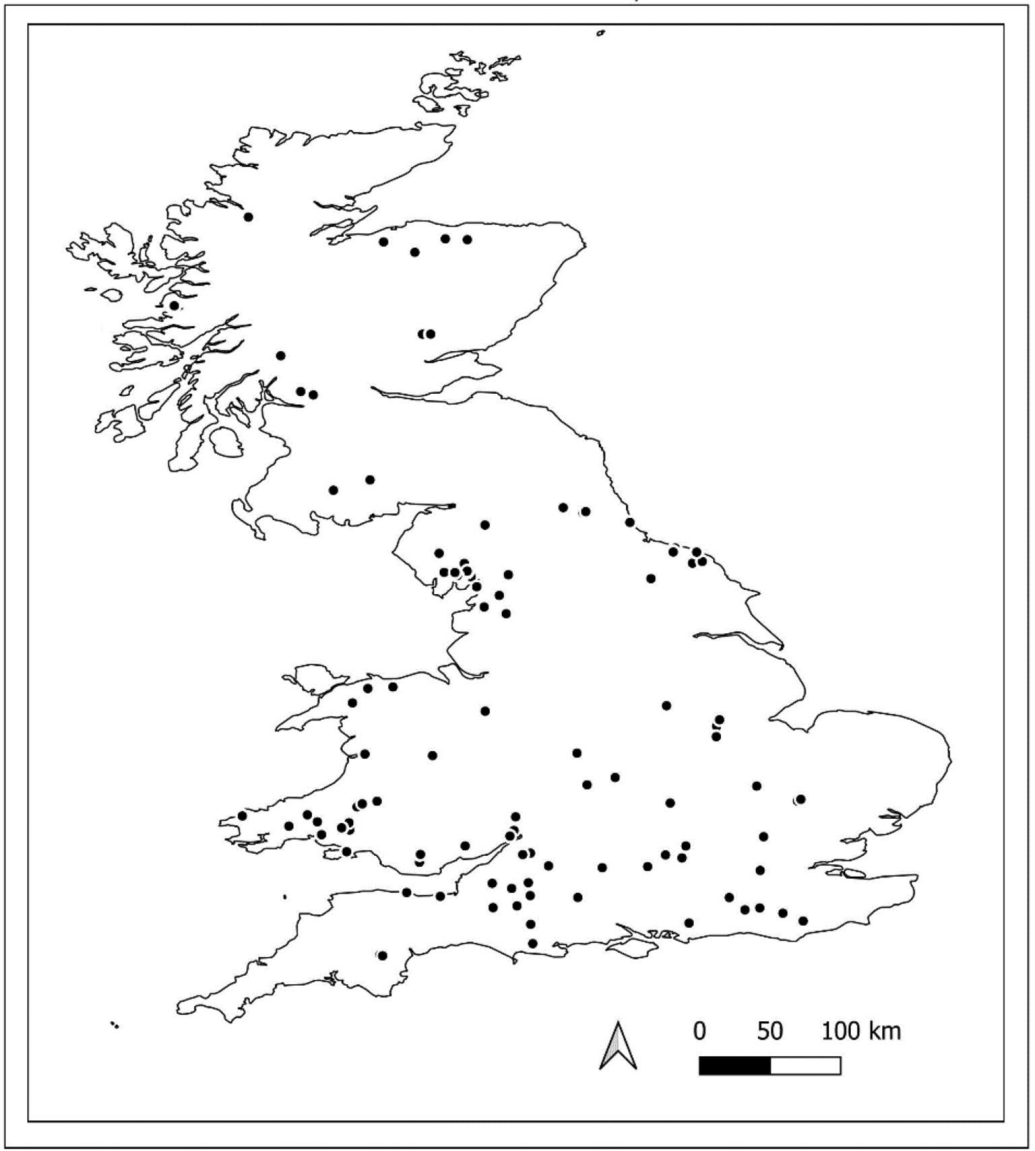
Geographic distribution of the 103 sampled sites for the 1971–2001 Woodland Survey of Great Britain. Location data from Kirby et al. (2013).

The Woodland Survey of Great Britain The Woodland Survey of Great Britain or ‘Bunce’ survey, first conducted in 1971 and repeated in 2001–2003 (Bunce and Shaw 1973; Wood et al. 2015; Kirby et al. 2005) sampled 103 semi‐natural woodland sites across England, Wales, and Scotland (Figure 1). The surveyed sites were selected from 2453 previously surveyed woodlands which had been organized into 103 groups using association analysis based on plant community similarities. The selected sites were identified as the most typical of their group (Wood et al. 2015). The sampled woodlands ranged in size from 4 to 312 ha, with a mean size of 31.8 ha, and were distributed over latitudes between 50.60 N and 57.76 N. At each site, 16 plots measuring 200 m^2^ were originally surveyed; data collected included vascular plant species, tree species, soil samples, and site descriptions. Vascular plants were recorded using nested quadrats covering the whole plot area. Abundance was measured as a percentage cover for each species over the entire plot. For the tree survey, diameter at breast height (DBH) and species were recorded for every individual tree over 5 cm diameter; smaller specimens were recorded in plot corners only. Trees were assigned to a size class ranging from 1 to 32, with classes set at 5 cm intervals, size class 1 starting at 1–5 cm DBH and size class 32 measuring 156–160 cm DBH. The available data also gave counts of individual species per size class in each surveyed plot.

### 2.2 Data Analysis—Replication Statement

The authors wish to understand how the extent of basal area of different tree species affects plant community functional trait expression and diversity in various woodlands. They measure community‐weighted means of plant functional traits and calculate diversity indices within 1274 plots across 103 woodland sites across Great Britain.

### 2.3 Data Cleaning and Criteria for Plot Inclusion

All data cleaning and processing was carried out using the R statistical software, unless otherwise stated (R Core Team 2024). This analysis focuses on the botanical and tree species survey data from the repeat Bunce survey of 2001–2003 (Smart, Wood, et al. 2024). Of the original 1648 plots surveyed in 1971, 1603 were available for this study. A total of 45 plots from the original survey were omitted either due to loss/change of the plot or because the plot was lacking either tree or plant data. Further plots were removed according to the following exclusion criteria:

1. Plots containing no vascular plants.
2. Plots where tree data consisted only of saplings of size class 1 (up to 5 cm DBH) were removed. Saplings of size class 1 were excluded from analysis on two grounds: firstly, saplings of this size were only counted in corners of plots in the original survey and secondly, it was assumed that saplings are less likely to be strong modifiers of soil conditions affecting established plants due to their less substantial rootzones and lower production of fine roots (Bardule et al. 2023). Trees have been shown to sequester carbon differentially according to age with implications for soil function (Ma et al. 2024), and the high mortality rate of saplings in general also equates to reduced opportunity to contribute to soil conditions.
3. Plots were removed if they were not primarily classified as ‘woodland’ by both the National Vegetation Classification System (NVC) (Rodwell 1991) and the Countryside Vegetation System (CVS), (Bunce et al. 1999). Previous work has shown that analyses were distorted by inclusion of ‘non forest’ plots (Corney et al. 2006). For this study, plots were classified into CVS and NVC types using the Modular Analysis of Vegetation Information System (MAVIS) Plot Analyzer (Smart 2000) using species and percentage‐cover data from each plot. Species recorded at less than 1% cover were excluded from the MAVIS analyses as the software does not recognize cover values below 1%. Removing species of less than 1% cover led to the loss of a further 182 plots which no longer contained sufficient botanical data for analysis.
4. Several plots were removed where the plant community was dominated by a non‐native invasive such as *Rhodondendron ponticum* L. or *Impatiens glandulifera* Royle. The exclusions affected only four plots across two sites, one upland oak and a lowland riparian valley woodland, with no implication for the representation of woodland types in the data.

After making the above omissions, the working dataset consisted of 1274 plots. Ash is rarely a dominant plot species and reached a minimum of 50% cover in 106 (8%) of plots at least 25% cover in 260 plots (20%) plots. Across all sites, ash was frequent or dominant (occurring in 41%–100% of site plots) in 45 (44%) of sites.

### 2.4 Quantifying the Extent of Ash and Other Tree Species in Sampled Plots

Counts of individual trees in plots were available in the survey dataset but deemed unreliable for this purpose as they would not account for tree size and maturity. The mean basal area of each species in a plot was used as a measure of species extent. Exact basal area quantities were not calculable due to diameter at breast height (DBH) measurements being organized into 5 cm size classes. Mean basal area was thus estimated using the mean DBH of each size class multiplied by the number of individuals of that species and size class in each plot. This step was repeated for the 6 most commonly occurring taxa alongside ash: *Corylus avellana* L. (Hazel), *Fagus sylvatica* L. (European Beech), *Quercus* spp. (Sessile or Pedunculate Oak), *Alnus glutinosa* (Alder), *Betula* spp. (Birches, all varieties), and *Acer pseudoplatanus* L. (Sycamore maple). Other species occurred too infrequently for meaningful analysis at a plot level.

### 2.5 Variable Selection—Controlling for Landscape and Environmental Influences

Nine explanatory parameters were included to account for environmental and edaphic variation; justification for their inclusion is provided below, while data sources are indicated in Table S1 in the Appendix and in the Data Availability Statement.

- Woodland area: The area of surveyed sites was not available from the publicly available survey data; however, patch‐size has been shown to be influential upon plant species diversity and community characteristics (Lomolino 2000; Godefroid and Koedam 2003). This parameter was calculated using QGIS (QGIS Development Team 2009) using data from the Woodland Survey of Great Britain.
- Edge‐effects: Using the same GIS data, distance from plots to woodland edges was calculated in QGIS to account for edge‐effects.
- Woodland type: Using the National Forest Inventory (Forestry Commission 2021), GIS data plots were categorized by woodland type into: ancient semi‐natural woodland (ASNW); plantation on ancient woodland site (PAWS); and secondary woodland (SEC).
- Soil Organic Matter (SOM): The composition of soil organic matter is influenced by the quality of plant and tree litter, and a two‐way relationship exists between SOM and plant community composition (Wardle et al. 2002; Ehrenfeld et al. 2005)
- Soil pH: Soil pH is a key moderator of plant community composition, directly controlling nutrient uptake and availability as well as the action of soil biota (Demeyer et al. 2001; Schaffers 2002)
- Management status: Management has a significant effect on species occurrence and distribution in woodlands (Mason 2007; Rackham 2008). The Woodland Survey data detailed whether woodlands were managed or unmanaged at the site level in most cases. Four sites (1, 5, 6 and 57) had missing data for this category. Management status was inferred at those sites by looking at the individual plot descriptions and recording the site as ‘managed’ where signs of woodland management were recorded.
- Latitude and Longitude: Latitude and longitude were included as explanatory variables to account for climatic differences between sites.
- 30‐year Mean Annual Rainfall: For a more localized climactic variable, the local mean‐annual rainfall from 1961 to 1990 was recorded at the site level, taken from the most proximate gauging station. The river flow and rainfall records of gauging stations are publicly available online from the Centre for Ecology and Hydrology's River Flow Archive (UKCEH 2024).
- Light availability—Mean Basal Area: Basal area is commonly used to estimate canopy cover and density in forestry and has been demonstrated to have usefulness as a metric for this purpose (McIntosh et al. 2012). As no data were available on light levels and canopy extent, mean basal area was used as a proxy of canopy cover.

### 2.6 Variable Selection—Plant Trait Data Selection and Data Sources

Nine plant functional traits were included as being most representative of key plant community functions (Table S2, Appendix). Additionally, the Shannon‐Wiener diversity index (Shannon 1948) was calculated for each plot using the Vegan package in R (Oksanen et al. 2013). Values for plant traits were obtained from the taxonomic, genetic and ecological vascular plant database resource compiled for RBG Kew (Henniges et al. 2022), which gathered plant trait data from a wide range of sources. Some additional trait data was taken from the Biolflor and LEDA databases (Kühn et al. 2004; Kleyer et al. 2008) accessed directly through the tr8 package for R (Bocci 2017). Selection of plant functional traits was based on the relative importance of traits to plant functional diversity. Traits were also selected if potentially influenced by the distinct nutrient cycling properties of ash trees or the enhanced light penetration under ash canopies.

Selected response trait groups:

- Ellenberg indicator values (EIVs) for Light, Moisture, Reaction/pH and 11 Nitrogen/Nutrients. The indicator values devised by Ellenberg (Hill et al. 1999) denote plant niche preferences in relation to seven key variables—light‐level, moisture/soil humidity, pH, nitrogen availability/fertility, temperature, salinity and continentality. Species are assigned an indicator value for each variable on an ordinal scale of 1–9 (12 for moisture). EIVs are some of the most widely used functional trait values in plant community analyses (Diekmann 2003)
- Specific leaf area (SLA), Mean Vegetation Height and Seed Mass. Specific leaf area, seed mass and vegetation height can explain the bulk of functional variation across plant species (Westoby 1998) and are a key measure in assessing the CSR strategy of plants (Grime 1977; Wilson et al. 1999)
- Diversity Indices the Shannon–Wiener index (Shannon 1948) was used here as a measure of species diversity and life form diversity using Raunkiaier's definitions relating to how perennating tissue is protected over winter (Raunkiaer 1934). Life form diversity was calculated from individual plot counts of the seven Raunkiaier lifeforms.

### 2.7 Calculation of Plant Trait Means and Working Dataset Creation

Plant and tree species names were homogenized between the Woodland Survey and plant trait datasets to ensure smooth merging of data. Plots were assigned individual plot ID numbers to assist with merging and data checking. Some tree species data was amalgamated for the purpose of these analyses. For example, *Quercus petraea* and *Quercus robur* (Sessile and Pedunculate Oak) data were aggregated—due to a high number of survey records where only the genus ‘Quercus’ is specified, plus high probability of confusion and hybridisation between the species. For identical reasons, records of *Betula pendula* and *Betula pubescens* were also amalgamated. The plant trait data was appended to the survey data at the plot level to enable calculation of community‐weighted means using R. Community‐weighted means are a commonly used measure of plant community composition and function (Garnier et al. 2015). Weighted mean values for plant traits were calculated for each plot, weighted by frequency. Frequency values for each species were calculated from the cover values supplied in the original data with species frequency as a percentage of the total summed species cover values for the plot.

Some botanical records from the original survey were ambiguous, for example: ‘*Cardamine hirsuta/flexuosa’*, in these cases traits were imputed as the mean of the two species except for traits where the two species values differed by more than one value level for Ellenberg traits and by more than 50% for seed mass, mean vegetation height and specific leaf area. Where trait differences exceeded these thresholds for ambiguous species, the traits values were not imputed. Several species were identified only to aggregate level and did not have corresponding trait values. Values were left blank where they could not be known or imputed and these species were excluded from the weighted mean calculations. Checks were made to ensure no excluded individuals comprised more than 5% cover in plots—with the exception of bramble aggregate species—and if this occurred, NA values were given for the trait value for that plot and it was excluded from the weighted mean calculations for those traits. Sample size was accordingly reduced for some traits due to the NA values, but always by less than 5%. The final database was transformed into wide format ready for analyses using the reshape function in R (R Core Team 2024).

### 2.8 Statistical Analysis and Descriptive Data

Linear mixed‐effects models were used to account for the hierarchical structure of our data, with plots nested within woodland sites. Site was included as a random effect to control for between‐site variation, with individual plots treated as the unit of replication. Models were fitted using restricted maximum likelihood with the lme4 package in R (Bates et al. 2015). For each response variable, we began with a maximal model containing all biologically plausible explanatory variables and selected interactions, then derived minimal adequate models through backward elimination based on parameter *p*‐values calculated using the lmerTest package (Kuznetsova et al. 2017).

We employed backward elimination because our primary goal was exploratory hypothesis testing rather than predictive modeling, for which this approach is well‐suited (Murtaugh 2009). We acknowledge that stepwise procedures can produce unstable model structures (Whittingham et al. 2006), therefore, we validated our model selection through comprehensive sensitivity analyses. For all response variables, we: (1) compared full and final models using AIC and BIC to confirm that backward elimination yielded better‐supported models; (2) refitted models using robust mixed‐model estimation (robustlmm package; Koller 2016) to assess sensitivity to outliers and non‐normality; (3) generated bootstrap confidence intervals (500 replicates) to evaluate parameter stability; and (4) refitted models after removing observations with extreme residuals (|standardized residual| > 2.5) to test whether conclusions were driven by extreme values.

The Mu‐MIn package for R (Barton 2025) was then used to compute R2 for each model. In order to minimize issues with multiple comparisons, and to constrain the study, we planned at the outset not to examine all interactions with explanatory variables. As the study aims to understand the relationship between ash and plant traits that are known to be strongly coupled with soil pH, an interaction with pH was included for all explanatory factors on all models except those looking at SLA, Ellenberg light and Ellenberg moisture traits. A fuller model was fitted for SLA involving interactions with latitude, longitude, rainfall and pH. In the case of Ellenberg light values, total plot mean basal area was included as an interaction term due to it being the most relevant parameter for light availability. Similarly, local mean annual rainfall 1961–1990 was included as an interaction term for models explaining variation in Ellenberg moisture values. Explanatory variables were initially measured on very different scales creating the potential to make model convergence problematic. Log transformations were applied to all explanatory variables except latitude and longitude; tree basal area was square root transformed for a marginally better distribution. Dependent variables were left untransformed except for SLA and seed mass which were log‐transformed to improve model fit. Model assumptions on normality and error distribution were checked graphically in sJplot package for R (Lüdecke 2024). The final minimal adequate models showed some non‐normality of residuals at the extreme ends of predicted values but otherwise met model assumptions adequately.

## 3 Results

### 3.1 Data Overview

Ash was present in 946 of the 1603 total plots (58%) and in 516 of the 1274 sampled plots used for this analysis (41%) and was the second most frequent and abundant species after oak, which was found in 625 of the sampled plots (49%). the third most frequent species, sycamore, occurred in 308 of the sampled plots (24%). Ash was fairly evenly distributed around the plot soil pH gradient; just under a third (160 of 516 plots) of ash‐containing plots were plots with pH measurements below pH 5. A further 139 plots containing ash were on weakly acid soils of pH 5–6.

Across all 1274 sampled plots, 503 species and/or aggregate species of vascular plant were recorded. Of these, 41 species have previously been described as ‘ash‐associated’ (Mitchell, Beaton, et al. 2014). Four of the ‘ash‐associated’ species sampled— *Primula elatior* (1 site, 5 plots), *Paris quadrifolia* (2 sites, 3 plots), *Convallaria majalis* (1 site, 2 plots) and *Daphne laureola* (2 sites, 3 plots) were found only in plots containing ash. A Fisher's Exact Test was applied to a 2 × 2 contingency table to assess whether plants classified as ash‐associated in the wider literature were more likely to be restricted to ash‐containing plots in the survey than non‐associated plants; the test found no significant association, with ash‐associated plants showing a slightly lower rate of plot exclusivity (9.8%) than non‐associated plants (12.3%), yielding an odds ratio of 0.77.

### 3.2 Model Results

**FIGURE 2:**
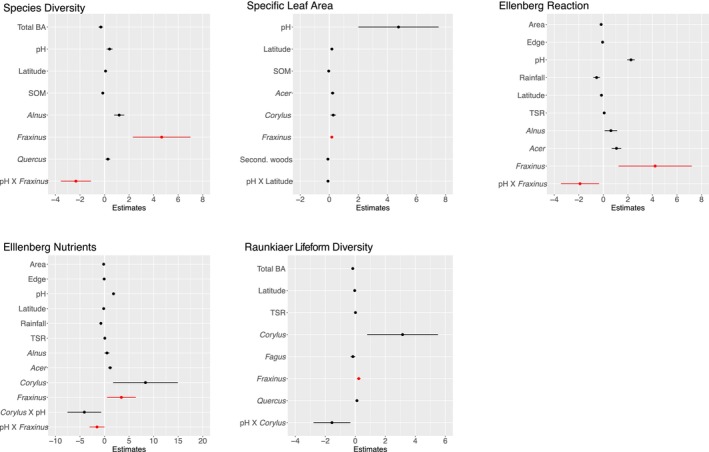
Five plant functional traits for ground flora significantly associated with extent of *Fraxinus* basal area in woodland plots surveyed in the Woodland Survey of Great Britain 2001–2003. Data are effect sizes with standard errors of significant predictors from mixed models, significant *Fraxinus* effects are highlighted in red. All tree genera refer to plot basal area for those taxa. Both diversity measures are Shannon Wiener indices, see text for full explanation of all functional traits and predictors. Second. woodland, secondary woodland; SOM, soil organic matter; Total BA, mean basal area; TSR, tree species richness.

Results of the linear mixed modeling showed a significant relationship between ash basal area and five of the nine plant functional response traits. Ash basal area was positively correlated with Shannon‐Weiner species diversity (*ß* = 2.54 ± 0.53, *p* < 0.0001); specific leaf area (*ß* = 0.12 ± 0.03, *p* < 0.0001); Ellenberg value for reaction (*ß* = 2.38 ± 0.67, *p* < 0.001); Ellenberg nutrients (*ß* = 1.75 ± 0.66, *p* < 0.01); and Raunkaier lifeform diversity (*ß* = 0.10 ± 0.03, *p* < 0.01) (Figure 2). No relationships were apparent between ash basal area and mean vegetation height or seed mass, nor with Ellenberg values for light and moisture. Regression model outputs for all nine traits and indices are presented in Tables S3–S11 in the Appendix.

We found that the effect sizes of ash in the above community analyses were large relative to other dominant tree species (Figure 2). For instance, ash basal area had the largest effect size on plot level plant species diversity and Ellenberg Reaction scores; ash also had the second largest effect size on Ellenberg Nutrients and Lifeform Diversity after hazel and the third greatest effect size on specific leaf area (Figure 2). Of all the tree species examined, only hazel had similarly strong associations with plant functional traits, particularly in terms of Specific Leaf Area and Ellenberg light scores (Appendix, Tables S4 and S10). Ellenberg reaction is a measure reflecting plant association with soil pH, and it is noteworthy that the main effect size of ash basal area on plot level Ellenberg reaction scores was greater than that of soil pH (albeit with greater variance).

**FIGURE 3:**
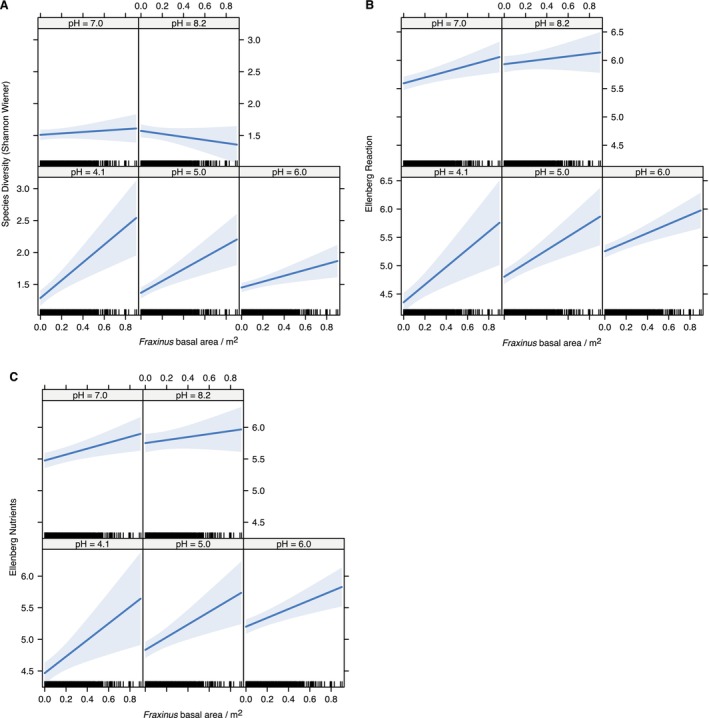
Interaction effect of *Fraxinus* mean basal area with pH on ground flora functional traits in plot level data from the Woodland Survey of Great Britain 2001–2003. Functional traits are: (A) species diversity (Shannon‐Wiener index); (B) community‐weighted mean Ellenberg Reaction score and (C) community‐weighted mean Ellenberg Nutrient score. Dark blue lines refer to effect size, while shaded blue areas are standard errors.

A key finding of the statistical modeling was the multiple robust interactions between ash basal and plot level soil pH (Figure 3). We identified significant negative interactions affecting plot level plant species diversity (*β* = −2.33 ± 0.62, *p* < 0.0001), Ellenberg reaction values (*β* = −1.89 ± 0.79, *p* < 0.01) and Ellenberg nutrients (*β* = −1.52 ± 0.77, *p* < 0.03). The interaction reflects contrasting relationships between ash abundance and these response variables across the pH gradient. For example, in the most acidic soils (pH 4.1), plot level diversity was approximately doubled across the observed range of ash basal areas (Figure 3A). In contrast, on neutral or basic soils, the association between diversity and ash abundance was flat or slightly negative. Ellenberg reaction and nutrient scores showed qualitatively similar patterns: strong positive association with ash basal area on acidic soils (pH 4.1–5.0) (Figure 3B,C). To put effect sizes in context, these Ellenberg scores range from 1 to 9; the high scores are attributed to plants associated with high pH (Ellenberg reaction) and high nutrient availability (Ellenberg nutrients). Ellenberg scores increased 1.5 points in the most acidic plots. Overall, this analysis demonstrates that ash has a much stronger association with diversity and functional variation in plant communities on acidic soils. On acidic soils in particular, where high ash abundance is linked to plant communities favoring high nutrient regimes and more alkaline conditions (Figure 3A–C).

### 3.3 Sensitivity Testing

Model comparison using Akaike information criteria (AIC) supported our models against the full models in every case. We employed robust mixed model analyses using the R package Robustlmm (Koller 2016) which confirmed that parameter estimates for some predictors differed over the range of 1.2%–28% from standard models across all response variables. The maximum difference for the ash predictor was 16.9%. Bootstrap confidence intervals (500 replicates) demonstrated that the majority of predictors were highly stable (36 of 39 total predictors showed 95% CIs excluding zero). We identified marginal instability in a small number of secondary predictors when extreme residuals were removed: mean basal area (diversity model), Alder basal area (Ellenberg nutrient model) and Beech basal area (lifeform diversity model) became non‐significant. Critically, ash basal area and its interactions remained robust across all five response variables (bootstrap CIs consistently excluding zero, trimmed data differences of 0.6%–12.4% and maintaining statistical significance in all sensitivity tests). This implies that our key ecological findings regarding ash effects on community structure and function are not driven by outliers, extreme observations, or violations of model assumptions.

## 4 Discussion

### 4.1 Is Ash a Functional Keystone in Woodland Ecosystems?

This analysis tested whether ash, and other dominant woody species, could potentially alter the fine‐scale composition of plant communities at the plot level. The study highlights that woody species composition can affect floral diversity and plant functional traits, analyzed as community weighted means. Importantly, this analysis supports the hypothesis that ash trees are functionally distinct among native trees in the UK and may contribute a particular set of conditions which provide certain plant community niches. Results showing a positive association of ash with community‐weighted means for nutrient availability, pH and diversity indices are compatible with previous studies investigating the plant communities found in ash‐frequent woods (Rodwell 1991; Mitchell, Hewison, et al. 2016; Thomas 2016). The results here suggest ash is ecologically unusual in terms of its strong impact on ground flora in relation to diversity and pH, an observation that follows from its known characteristics in terms of light penetration and nutrient turnover (Scheu and Schauermann 1994; Hagen‐Thorn et al. 2004; Langenbruch et al. 2012; Thomas 2016). There is clearly some functional overlap between tree species in relation to plant variation. Alder and sycamore abundance also correlated with five of the nine studied traits although generally with substantially reduced effect sizes compared to ash. Sensitivity analyses also indicate that the alder result may be confounded by other site factors.

Ash shows a consistently larger effect size upon plant species diversity and Ellenberg reaction scores than the other dominant broad‐leaved tree species examined for most response variables. However, the results also point towards potentially distinct functional effects of Hazel (*C. avellana*) for specific leaf area (SLA), Ellenberg nutrient and light community scores and Raunkiaier lifeform diversity. There was no collinearity between the presence of ash and hazel in the sampled plots. Hazel produced a considerably larger effect size than ash on Ellenberg nutrient scores and Raunkiaer lifeform diversity with a similar negative interaction with pH, suggesting the structural effect of both species is enhanced in more acidic conditions. Correlations involving hazel may be related to its usual role as an understorey species and the association of hazel coppice with structural heterogeneity and the biodiversity associated with past or recent coppice management. However, the functional status of hazel trees in comparison to other common woodland species warrants further exploration.

The positive association between ash, hazel and sycamore and higher community SLA is interesting, but should be interpreted cautiously. Community specific leaf area is considered one of the most fundamental plant functional traits (Westoby 1998; Shipley 2002; Dahlgren et al. 2006) which has been shown to interact strongly with soil pH, nutrient and water availability, light levels and climatic gradients (Wright et al. 2004; Meziane and Shipley 1999). Lower light conditions are associated with higher SLA (Dwyer et al. 2014) so canopy species creating more shade such as sycamore might be expected to drive community SLA upwards. SLA is also driven by nutrient availability, but ash, hazel and sycamore all frequently occupy fertile, more base‐rich sites. The observed relationships are correlative and the SLA signal may partly or wholly reflect underlying site conditions rather than a direct species‐driven effect, but if canopy effects on plant community SLA are present, they may be driven by different, species‐specific mechanisms.

Previous studies examining the 1971 Woodland Survey data determined that geo‐climactic factors are the primary influences upon woodland plant community assemblages. Corney et al. (2006) concluded that climate and soil pH gradients explained much variation in plant communities and these effects were modified by local factors such as deer browsing, woodland boundary and management. In that study, tree species composition was found to be a significant, yet relatively small, driver of plant community variation. However, it was noted that important fine‐scale relationships between woody species and plant community composition may be obscured by pH and climate gradients. This study, with a focus on tree species as floristic drivers and variation within, rather than between, woodlands, does not contradict earlier work on the drivers of ground flora composition in UK woodlands, but adds complexity to current understanding.

### 4.2 Are Ash Trees Uniquely Involved in Critical Plant–Soil Feedbacks?

The existence of plant–soil feedbacks has been acknowledged for some time (Zinke 1962; Bardgett and Wardle 2010; Mangan et al. 2010). Soil variables under different plant species have been shown to vary by 41% between species treatments (Waring et al. 2015). The positive relationships found here between ash and diversity indices, and between ash and community Ellenberg scores for reaction and nutrients are consistent with the known preference of ash with higher pH, base‐rich soils. However, a key unexpected result of this study was the interaction effect between soil pH and ash prevalence in plant community diversity and functional variation. Ash basal area had greater impact on three of our focal traits at lower pH values. The interpretation here is that ash either offsets the effects of acidic soils or soil pH moderates the effect of ash prevalence. The interaction of ash with pH on plant community properties implies and increased structural influence of ash upon woodland plant communities in lower pH soils with which it is not so preferentially associated.

Much work forecasting the impacts of ash loss has focused on ash‐frequent woodlands; therefore, potential vegetation changes relating to the fine‐scale function of ash trees in other woodland habitats may be underappreciated. The residual and random variance in the model examining Ellenberg nutrient scores were low. The relatively low contribution of the random effect of ‘site’ (22%) suggests nutrient availability is more subject to belowground processes than aboveground factors such as disturbance, management and pollution. This inference is supported by studies which show potentially dramatic long‐term effects of changes to nutrient regimes following alteration in leaf litter quantity and/or quality (Cole 1995; Sayer 2006). This study focusing on ash effects at the plot level supports the probability that some altered nutrient functioning in response to the loss of ash may occur at very localized scales, regardless of total ash canopy extent.

### 4.3 Could Loss of Ash Cause Dramatic Changes in Forest Soils and Plant Communities?

It is important to emphasize the most immediate and potentially substantial effects of ash loss on forest ecosystems will be via the structural changes from canopy gap creation previously discussed. The association of four ‘ash associated’ species with ash plots, while not statistically significant in this dataset, may still be ecologically meaningful as their association with ash‐frequent woods may exist within a broader context involving site structural or edaphic factors, rather than spatial proximity to ash trees and may justify further investigation in the case of ash‐associated plants of conservation concern such as *P. elatior* which is ‘near threatened’ in the UK (Stroh et al. 2025).

The finer functional impacts of widespread ash loss on soil nutrient cycling and plant communities as a result of ash dieback may involve significant time lags. Following large losses of other Fraxinus species in the US due to the Emerald Ash Borer (EAB) (*Agrillus planiplennis*, Fairmaire.*)*, several forest‐floor ecosystem impacts related to changed nutrient regimes have been reported; successional shifts in soil communities (Ricketts et al. 2018), changes to stream nutrient regimes and aquatic invertebrates (Kreutzweiser et al. 2020), and the simplification of arachnid food webs due to changed soil biota (Michalko et al. 2021). Ash is also distinct from many other tree species below ground as well as above in forming arbuscular mycorrhizal (AM) associations rather than the more common ectomycorrhizal partnerships (Thomas 2016). It is not known how high losses of ash may impact AM functioning within individual forest patches and related impacts on nutrient availability.

The functional impacts of widespread ash loss on soil nutrient cycling and plant communities as a result of ash dieback may involve significant time lags. Ash dieback has not yet eliminated ash entirely from many affected areas. The recent repeat of the ‘Bunce’ woodland survey reported 21% of original survey plots were affected by ash dieback, but widespread total loss of ash from plots was not reported (Smart, Walker, et al. 2024). No significant differences in species richness, diversity or community weighted Ellenberg light values were found when baseline vegetation data from Norwegian forest stands collected before ash dieback arrived was compared with new data recorded 7–11 years after pathogen outbreak (Schei et al. 2024). However, Ellenberg nitrogen and reaction value comparisons were not reported in that study and ash was still present in 52 out of 58 original ash plots. In functional terms, thinning of ash canopy from ash dieback infections will increase light levels reaching the understory‐ this community is already well‐suited to high light levels relative to other woodlands. Potentially this could slow the response of plant communities to ash dieback. Impacts of ash loss on soil nutrient cycling and plant community traits may be cryptic until after ash cover has been substantially or completely eliminated.

### 4.4 Study Limitations and Future Prospects

A major limitation of this study is that it is survey based and correlational. This type of study is also vulnerable to the influence of confounding factors. Importantly, colinearity between edaphic features and ash abundance could obscure ecologically functional relationships. The data available capture soil pH but ash responds strongly to other soil characteristics such as nitrogen availability (Gordon 1964), which might also drive some of the observed associations. Nevertheless, the patterns found here suggest valuable hypotheses for future work, particularly in terms of the interaction of ash and soil pH. Key interacting variables missing from this analysis are grazing intensity and pollution pressure which are likely to create differential contexts across woodlands, modifying any functional contributions of ash in woodland ecosystems. The latest Bunce re‐survey data suggest a prevalence of pollution‐related increases in plant community Ellenberg N scores; it also found that grazing pressure increases species richness of ground flora in ash‐dieback affected woodland plots, potentially by mediating the impacts of competitor species (Smart, Walker, et al. 2024).

Also not considered in this analysis were the impacts of previous woodland management and stochastic disturbance which potentially long‐ lasting in forest environments (Kirby et al. 2005; Hopkins and Kirby 2007; Rackham 2008; Ramalho et al. 2018). In terms of the future management of woodlands affected by ash dieback, recent work has emphasized the robust regeneration potential of ash and the high health of saplings (Coker et al. 2019; Morris and Davies 2025). There is increasing evidence for a disease resistant fraction of trees (Coker et al. 2019; Carroll and Boa 2024; Seidel et al. 2024). Together this suggests that facilitating natural recovery is a sensible way forward for ash woodlands. Any management interventions that can encourage regeneration, eg by suppressing rabbit grazing, might therefore be beneficial. Monitoring of recruitment near healthy individuals would provide valuable data in the future. Given the additional evidence for status of ash as a keystone tree species in this study, management of ash dieback by replanting with other species might have drawbacks where there is clear regeneration potential.

## 5 Conclusions

This study found ash trees are strongly associated with species diversity and plant functional traits in the woodland communities; association also varied with pH conditions, with evidence of interaction between the influence of ash and local soil pH. Ash basal area was significantly correlated with a higher number of tested functional traits than most other broadleaved species and commonly yielded larger effect sizes, reinforcing the ecological distinctiveness attributed to ash. The UK is due to experience unprecedented losses of this prominent and potentially keystone species over the coming decades. The possibility that ash trees influence plant community composition more strongly on lower pH soils, as these findings suggest, implies that vascular plant composition in woodlands may yet significantly change in less predictable ways following significant loss of ash.

## Author Contributions

**Melanie Roach:** conceptualization (lead), formal analysis (lead), investigation (lead), methodology (equal), visualization (lead), writing – original draft (lead), writing – review and editing (supporting). **Ben Raymond:** data curation (lead), methodology (supporting), supervision (lead), visualization (supporting), writing – review and editing (lead).

## Conflicts of Interest

The authors declare no conflicts of interest.

## Supporting information


**Table S1:** Explanatory variables for multiple regression analysis covering woodland features, soil properties, climate and tree species. Woodland Survey of Great Britain (WSGB) (Wood et al. 2015) data are publicly available from the Centre of Ecology and Hydrology, UK. National Forest Inventory (NFI) (Forestry Commission 2021) data are held by the Forestry Commission. Tree basal area was calculated for the seven dominant canopy taxa: *Acer pseudoplatanus* (Sycamore), *Alnus glutinosa* (Alder), *Betula* spp. (Birches), *Corylus avellana* (Hazel), *Fagus sylvatica* (Beech), *Fraxinus excelsior* (Ash), *Quercus* spp. (Oaks).
**Table S2:** Nine plant functional traits and diversity indices used as response data in multivariate regression analysis of plant community trait expression in surveyed plots.
**Table S3:** Species diversity (Shannon‐Wiener Index).
**Table S4:** Specific Leaf Area.
**Table S5:** Community‐Weighted Mean Ellenberg Reaction Score.
**Table S6:** Community‐Weighted Mean Ellenberg Nutrients Score.
**Table S7:** Raunkiaer Lifeform Diversity (Shannon Wiener index).
**Table S8:** Mean Vegetation Height.
**Table S9:** Seed mass.
**Table S10:** Ellenberg Light Score.
**Table S11:** Ellenberg Moisture Score.

## Data Availability

All data used in this paper are publicly available. Survey data from ‘The Woodland Survey of Great Britain’ https://doi.org/10.5285/42c203c8‐44de‐40e2‐a694‐b1e8cbd4c8e1 are freely available online from the UKCEH Environmental Information Data Centre as is the plant trait database: ‘A taxonomic, genetic and ecological data resource for the vascular plants of Britain and Ireland’ https://doi.org/10.5285/9f097d82‐7560‐4ed2‐af13‐604a9110cf6d. Compiled data and analysis code are available from Figshare with the permanent doi:10.6084/m9.figshare.31791949.
