# Supplementary material for: European Ash (Fraxinus excelsior) as a Functional Keystone Species Shaping Woodland Flora in the UK
Source: Ecol Evol. 2026 Apr 2;16(4):e73356. doi: 10.1002/ece3.73356 (PMC13045497; doi:10.1002/ece3.73356)
Supplement: Supplementary file 1 — Table S1: Explanatory variables for multiple regression analysis covering woodland features, soil properties, climate and tree species. Woodland Survey of Great Britain (WSGB) (Wood et al. 2015) data are publicly available from the Centre of Ecology and Hydrology, UK. National Forest Inventory (NFI) (Forestry Commission 2021) data are held by the Forestry Commission. Tree basal area was calculated for the seven dominant canopy taxa: Acer pseudoplatanus (Sycamore), Alnus glutinosa (Alder), Betula spp. (Birches), Corylus avellana (Hazel), Fagus sylvatica (Beech), Fraxinus excelsior (Ash), Quercus spp. (Oaks). Table S2: Nine plant functional traits and diversity indices used as response data in multivariate regression analysis of plant community trait expression in surveyed plots. Table S3: Species diversity (Shannon‐Wiener Index). Table S4: Specific Leaf Area. Table S5: Community‐Weighted Mean Ellenberg Reaction Score. Table S6: Community‐Weighted Mean Ellenberg Nutrients Score. Table S7: Raunkiaer Lifeform Diversity (Shannon Wiener index). Table S8: Mean Vegetation Height. Table S9: Seed mass. Table S10: Ellenberg Light Score. Table S11: Ellenberg Moisture Score. [file ECE3-16-e73356-s001.docx]

**Appendix- supplementary tables**

**Table S1**: Explanatory variables for multiple regression analysis covering woodland features, soil properties, climate and tree species. Woodland Survey of Great Britain (WSGB) (Wood, et al, 2015) data are publicly available from the Centre of Ecology and Hydrology, UK. National Forest Inventory (NFI) (Forestry Commission, 2021) data are held by the Forestry Commission. Tree basal area was calculated for the seven dominant canopy taxa: *Acer pseudoplatanus* (Sycamore), *Alnus glutinosa* (Alder), *Betula* spp. (Birches), *Corylus avellana* (Hazel), *Fagus sylvatica* (Beech), *Fraxinus excelsior* (Ash), *Quercus* spp (Oaks).

| **Variable** | **Units** | **Description** | **Source** |
| --- | --- | --- | --- |
| **Environmental:**  Wood area | m^2^ | Entire conjoined woodland area, including coniferous (buffer set to 7m to account for forestry tracks and minor roads) | Calculated with QGIS using shapefile data from WSGB and NFI. |
| Distance to edge | metres | Distance from plot to nearest forest edge | Calculated with QGIS using shapefile data from WSGB and NFI. |
| Management status | categorical | managed’ or ‘unmanaged’ | WSGB |
| Woodland age | categorical | ‘ancient’, ‘secondary’ or ‘plantation on ancient woodland’ (PAWS) | NFI |
| **Climactic:**  Mean annual rainfall | millimetres | Average annual rainfall 1961-1990 | National river flow archive (Centre for Ecology and Hydrology) |
| Longitude | decimal | Plot co-ordinate | Calculated from plot easting/northing data from WSGB (provided upon request by the Centre for Ecology and Hydrology) |
| Latitude | decimal | Plot co-ordinate |  |
| **Edaphic:**  Soil pH | pH scale | Plot soil pH taken from a mean of sample | WSGB |
| Soil organic matter | % | Plot organic matter percentage |  |
| **Composition**  Tree species richness  Mean basal area: |  | Plot species count – all tree and shrub species in plot | WSGB |
|  | m^2^ | Calculated from survey data on individual counts of tree species at size classes of 5cm intervals of diameter at breast height (DBH) | WSGB |

**Table S2:** Nine plant functional traits and diversity indices used as response data in multivariate regression analysis of plant community trait expression in surveyed plots.

| **Plant trait data** | **Units** | **Source** |
| --- | --- | --- |
| Species Diversity (Shannon-Wiener) | Continuous values from 0-5 | Vegan v1.8-5J Oksanen (2017) |
| Specific Leaf Area | mm^2^ mg-^1^ | Henniges et al (2022) |
| Mean vegetation height | cm | Henniges et al (2022) |
| Seed mass | mg | Henniges et al (2022), LEDA (Kleyer et al 2008) |
| Ellenberg Light | Community-weighted mean from scores of 2-9 | Henniges et al (2022) |
| Ellenberg Nutrients | Community-weighted mean from scores of 2-9 | Henniges et al (2022) |
| Ellenberg Moisture | Community-weighted mean from scores of 2-9 | Henniges et al (2022) |
| Ellenberg Reaction | Community-weighted mean from scores of 2-9 | Henniges et al (2022) |
| Raunkiaer Lifeform Diversity (Shannon-Wiener). | Continuous values from 0-5 | Vegan v1.8-5J (Oksanen, 2017)  and Henniges et al (2022) |

**Tables S3-11.** Estimated regression parameters, standard errors, t-values and significance for the linear mixed effects models explaining variation of nine plant functional traits between plots characterised in the Woodland Survey of Great Britain. Tables show only statistically significant relationships to predictor variables. Tree genera listed as predictors refer to plot basal area for those taxa. Interactions by separating terms with colons, eg pH:*Fraxinus*. SOM refers to Soil organic matter. Significance * *P* < 0.05, ** < 0.01, *** <0.001

**Table S3. Species diversity (Shannon-Wiener Index)**

| **Random Effects:** | |  |  |
| --- | --- | --- | --- |
|  | Variance | Std.Dev. |  |
| Site | 0.108 | 0.3287 |  |
| Residual | 0.2607 | 0.5105 |  |
|  | |  |  |
| **Fixed effects:** | |  |  |
|  | Estimate | SE | *t* |
| (Intercept) | -3.420 | 1.101 | -3.106** |
| *Fraxinus* | 4.658 | 1.196 | 3.894*** |
| pH:*Fraxinus* | -2.332 | 0.623 | -3.746*** |
| *Alnus* | 1.195 | 0.211 | 5.663*** |
| pH | 0.412 | 0.122 | 3.368*** |
| Total Basal Area | -0.302 | 0.090 | -3.372*** |
| *Quercus* | 0.271 | 0.097 | 2.81** |
| SOM | -0.143 | 0.034 | -4.171*** |
| Latitude | 0.087 | 0.020 | 4.372*** |

**Table S4. Specific Leaf Area**

| **Random Effects:** | |  |  |
| --- | --- | --- | --- |
|  | Variance | Std.Dev. |  |
| Site | 0.021 | 0.145 |  |
| Residual | 0.039 | 0.198 |  |
|  | |  |  |
| **Fixed effects:** | |  |  |
|  | Estimate | SE | *t* |
| (Intercept) | -6.435 | 2.583 | -2.492* |
| pH | 4.763 | 1.406 | 3.387*** |
| *Corylus* | 0.274 | 0.094 | 2.904** |
| *Acer* | 0.231 | 0.061 | 3.804*** |
| Latitude | 0.181 | 0.049 | 3.687*** |
| *Fraxinus* | 0.177 | 0.046 | 3.821*** |
| pH:Latitude | -0.087 | 0.027 | -3.261** |
| Secondary Woods | -0.093 | 0.021 | -4.361*** |
| SOM | -0.038 | 0.014 | -2.81** |

**Table S5. Community-Weighted Mean Ellenberg Reaction Score**

| **Random Effects:** | |  |  |
| --- | --- | --- | --- |
|  | Variance | Std.Dev. |  |
| Site | 0.274 | 0.523 |  |
| Residual | 0.413 | 0.643 |  |
|  | |  |  |
| **Fixed effects:** | |  |  |
|  | Estimate | SE | *t* |
| (Intercept) | 14.062 | 1.757 | 8.005*** |
| *Fraxinus* | 4.218 | 1.520 | 2.774** |
| pH | 2.252 | 0.157 | 14.365*** |
| pH:*Fraxinus* | -1.899 | 0.791 | -2.401* |
| *Acer* | 1.069 | 0.198 | 5.391*** |
| *Alnus* | 0.614 | 0.261 | 2.354* |
| Avg rainfall | -0.549 | 0.138 | -3.987*** |
| Wood area | -0.181 | 0.046 | -3.929*** |
| Latitude | -0.158 | 0.032 | -4.992*** |
| Tree Species Richness | 0.070 | 0.014 | 4.912*** |
| Edge distance | -0.063 | 0.024 | -2.693** |

**Table S6. Community-Weighted Mean Ellenberg Nutrients Score**

| **Random Effects:** | |  |  |  |
| --- | --- | --- | --- | --- |
|  | Variance | Std.Dev. |  |  |
| Site | 0.281 | 0.530 |  |  |
| Residual | 0.393 | 0.627 |  |  |
|  | |  |  |  |
| **Fixed effects:** | |  |  |  |
|  | Estimate | SE | *t* |  |
| (Intercept) | 16.670 | 1.768 | 9.430*** |  |
| *Corylus* | 8.348 | 3.356 | 2.487* |  |
| *Fraxinus* | 3.442 | 1.487 | 2.315* |  |
| pH | 1.835 | 0.159 | 11.576*** |  |
| *Acer* | 1.143 | 0.194 | 5.885*** |  |
| *Alnus* | 0.503 | 0.255 | 1.973* |  |
| Tree Species Richness | 0.061 | 0.014 | 4.349*** |  |
| Edge distance | -0.074 | 0.023 | -3.23** |  |
| Latitude | -0.169 | 0.032 | -5.287*** |  |
| Wood area | -0.172 | 0.046 | -3.717*** |  |
| Rainfall | -0.740 | 0.139 | -5.332*** |  |
| pH:*Fraxinus* | -1.525 | 0.773 | -1.971* |  |
| pH:*Corylus* | -4.111 | 1.750 | -2.349* |  |

**Table S7. Raunkiaer Lifeform Diversity (Shannon Wiener index)**

| **Random Effects:** | |  |  |
| --- | --- | --- | --- |
|  | Variance | Std.Dev. |  |
| Site | 0.0167 | 0.13) |  |
| Residual | 0.056 | 0.237 |  |
|  | |  |  |
| **Fixed effects:** | |  |  |
|  | Estimate | SE | *t* |
| (Intercept) | 3.018 | 0.434 | 6.957*** |
| *Corylus* | 3.152 | 1.202 | 2.621** |
| pH:*Corylus* | -1.555 | 0.626 | -2.483* |
| *Fraxinus* | 0.231 | 0.060 | 3.857*** |
| Tree Basal Area | -0.169 | 0.045 | -3.714*** |
| *Fagus* | -0.162 | 0.078 | -2.083* |
| *Quercus* | 0.110 | 0.046 | 2.395* |
| Latitude | -0.039 | 0.008 | -4.807*** |
| Tree Species Richness | 0.012 | 0.005 | 2.246* |

**Table S8. Mean Vegetation Height**

| **Random Effects:** | |  |  |
| --- | --- | --- | --- |
|  | Variance | Std Dev |  |
| Site | 0.0023 | 0.0485 |  |
| Residual | 0.0090 | 0.0948 |  |
|  |  |  |  |
| **Fixed effects:** | |  |  |
|  | Estimate | SE | *t* |
| (Intercept) | 1.279 | 0.176 | 7.26*** |
| *Betula* | -0.012 | 0.005 | 2.22* |
| pH:*Betula* | -0.081 | 0.016 | -2.15* |
| Acer | -0.010 | 0.003 | 3.54*** |
| Tree Basal Area | -0.036 | 0.014 | -5.09*** |
| *Quercus* | 0.041 | 0.014 | 2.76** |
| SOM | 0.104 | 0.029 | 2.84** |
| Avg rainfall | 0.764 | 0.344 | -2.57* |
| pH:SOM | 0.048 | 0.017 | -2.60** |
| Wood area | -0.427 | 0.199 | -2.55* |
| Latitude | -0.020 | 0.008 | -2.98** |

**Table S9. Seed mass.**

| **Random Effects:** | |  |  |
| --- | --- | --- | --- |
|  | Variance | Std.Dev. |  |
| Site | 0.4283 | 0.6544 |  |
| Residual | 0.6566 | 0.8103 |  |
|  |  |  |  |
| **Fixed effects:** | |  |  |
|  | Estimate | SE | *t* |
| (Intercept) | 9.346 | 2.158 | 4.33*** |
| pH | 1.558 | 0.310 | 5.02*** |
| Longitude | -0.630 | 0.233 | -2.70** |
| Managed woods | -0.402 | 0.147 | -2.73** |
| pH:Longitude | 0.324 | 0.128 | 2.53* |
| Total Basal Area | 0.280 | 0.121 | 2.32* |
| Latitude | -0.219 | 0.040 | 5.54*** |
| TSR | 0.0792 | 0.019 | 4.25*** |

**Table S10. Ellenberg Light Score.**

| **Random Effects:** | |  |  |
| --- | --- | --- | --- |
|  | Variance | Std.Dev. |  |
| Site | 0.1696 | 0.4119 |  |
| Residual | 0.2878 | 0.5365 |  |
|  | |  |  |
| **Fixed effects:** | |  |  |
|  | Estimate | SE | *t* |
| (Intercept) | 7.843 | 0.233 | 33.71*** |
| Total Basal Area | -3.512 | 1.043 | -3.37*** |
| *Corylus* | -3.222 | 0.567 | -5.68*** |
| Total Basal Area:*Corylus* | 3.099 | 0.663 | 4.67*** |
| pH | -1.176 | 0.122 | -9.64*** |
| Alnus | 0.849 | 0.223 | 3.80*** |
| Total Basal Area:*Quercus* | 0.570 | 0.134 | 4.27*** |
| Acer | -0.449 | 0.172 | -2.61** |
| Total Basal Area:Rainfall | 0.410 | 0.150 | 2.73** |
| Managed woods | 0.209 | 0.093 | 2.24* |
| TSR | -0.052 | 0.012 | -4.20*** |

**Table S11. Ellenberg Moisture Score**

| **Random Effects:** | |  |  |
| --- | --- | --- | --- |
|  | Variance | Std.Dev. |  |
| Site | 0.08361 | 0.2891 |  |
| Residual | 0.1296 | 0.36 |  |
|  |  |  |  |
| **Fixed effects:** |  |  |  |
|  | Estimate | SE | *t* |
| (Intercept) | 7.48895 | 1.18695 | 6.309*** |
| *Alnus* | 1.2461 | 0.146 | 8.535*** |
| Longitude | 0.92048 | 0.39673 | 2.32* |
| pH | 0.61442 | 0.0831 | 7.394*** |
| Avg rainfall | -0.43037 | 0.1722 | -2.499* |
| *Fagus* | -0.34926 | 0.11051 | -3.16** |
| *Quercus* | -0.19073 | 0.05763 | -3.31*** |
| Longitude:Avg rainfall | -0.13781 | 0.05631 | -2.447* |
